# Follow-up after successful endoscopic therapy for early Barrett’s neoplasia: Is it time to talk money yet?

**DOI:** 10.1055/a-1931-3841

**Published:** 2022-10-17

**Authors:** E. P.D. Verheij, S. N. van Munster, R. E. Pouw

**Affiliations:** 1 Amsterdam UMC location University of Amsterdam, Gastroenterology and Hepatology, Amsterdam, The Netherlands; 2 Sint Antonius Hospital, Gastroenterology and Hepatology, Nieuwegein, Netherlands

 Endoscopic eradication therapy (EET) using endoscopic resection followed by radiofrequency ablation (RFA) has proven to be a safe and highly effective treatment for patients with Barrett’s-related neoplasia. Recently, long-term follow-up studies have reported a low risk for development of neoplastic recurrences in patients who were successfully treated and achieved complete eradication of dysplasia and intestinal metaplasia (CE-IM) 1 2 . This has put a new light on the discussion regarding surveillance protocols after EET. Currently, these surveillance protocols are based on expert opinion and derive from the era when endoscopic resection was performed with surveillance of the remaining Barrett’s esophagus (BE) segment, instead of pursuing complete eradication of all BE using RFA. The quest for the optimal surveillance protocol after successful EET is ongoing, and with the recent long-term follow-up studies, current strategies will likely be subject to change.

An important issue when considering surveillance is its cost-effectiveness. Menon et al. performed an interesting cost-effectiveness analysis with the aim to determine the most optimal follow-up approach in current clinical care 3 . The surveillance protocols used in the analysis were the commonly used guidelines of the American College of Gastroenterology (ACG), the “UK’’ strategy (used in many centers in the United Kingdom) and the new “Cotton” strategy 4 5 6 . The ACG-strategy, based on the ACG-guideline, suggests 3-monthly endoscopic surveillance in the first year after achieving CE-IM, 6-monthly endoscopies in the second year, followed by annual surveillance thereafter for patients with high-grade dysplasia (HGD) or intramucosal cancer (IMC) as pre-RFA diagnosis. For patients with low-grade dysplasia (LGD) or indefinite for dysplasia before RFA, the ACG suggests surveillance every 6 months in the first year, followed by annual surveillance thereafter 4 . The “Cotton” strategy was developed using models to predict the risk of recurrent dysplasia after RFA therapy. For patients with LGD pre-RFA, they suggest surveillance after 1 and 3 years, while patients with HGD or IMC pre-RFA should have surveillance after 3, 6 and 12 months, and annually thereafter 5 . Another strategy, the so-called “UK” strategy, suggests 3-monthly surveillance in the first year after CE-IM, followed by surveillance every 6 months in the second year and annual endoscopic surveillance thereafter, irrespective of pre-RFA diagnosis 6 . Menon et al created a Markov model of patients with successful EET, assuming a 0.02-point estimate for recurrent dysplasia after 1 year of follow-up, increasing to 0.06 after 7 years of follow-up. Patients entered at age 50 and underwent endoscopic follow-up until age 90 or death, whichever occurred first. Interestingly, the most rigorous strategy, the ACG strategy, appeared most cost-effective.

Incidence of recurrent disease and its early detection plays a key role in determining surveillance protocols. Previous studies have shown that the annual risk for recurrent neoplasia is low, around 1 % to 2 % 1 2 7 . However, the definition of what a recurrence comprises is still not unanimous among endoscopists. In our opinion, there is a difference in clinical relevancy when considering “clinically non-significant” recurrence of visible BE without dysplasia and recurrent IM from random biopsies in a normal appearing cardia, versus “clinically significant” recurrence of dysplasia requiring (endoscopic) retreatment. Also, when discussing clinical relevancy of a recurrence and its therapeutic consequences, we may need to take into account the overall health and life expectancy of the patient as well, instead of using a one-size-fits-all approach. In the end, the aim of endoscopic therapy and surveillance thereafter should be to prevent symptomatic disease or progression to disease stages that exceed the boundaries for endoscopic treatment. So, one may question whether an asymptomatic recurrence of dysplasia is clinically relevant for all patients, since not all patients will live long enough to progress to advanced cancer. In our opinion, recurrence of non-dysplastic BE or IM, therefore, should not be guiding in defining FU protocols.

Instead of using the one-size-fits-all approach, shouldn’t we better try to individualize post-EET surveillance? On one hand, we should try to identify subgroups of patients with a high risk for recurrence, who require more frequent follow-up, versus the majority of patients with a very low risk for recurrence. Prior studies have shown that patients with increasing BE length, more treatments, worse baseline histology, and younger age are found to be more prone to develop recurrences after treatment than other patients 8 9 . Identifying a subgroup of patients with a high risk for recurrence that may benefit from more frequent FU may allow us to minimize the frequency of FU endoscopies in the vast majority of patients.

But is the incidence of recurrence the only factor we should take into account when establishing our protocols? A patient’s life expectancy also plays an important role, since follow-up is initiated to detect asymptomatic disease at an early stage and to prevent progression to clinically relevant disease in the future. Prior studies reported high mortality rates from causes other than recurrent esophageal cancer during follow-up 1 10 . Of note, a recent study reported that 8 % of patients died from unrelated causes during a median follow-up of 4 years, while the current study of Menon et al. assumed that 8 % of patients would die during a follow-up period of 40 years 1 3 . This underestimation of the risk of other-cause mortality, or in fact, an overestimation of a patient’s life expectancy, may affect outcomes of a cost-effectiveness analysis. Furthermore, in the current model by Menon et al., the assumption was made that all patients up to age 90 years underwent endoscopic follow-up and were fit enough to receive endoscopic therapy for a recurrence 3 . We think that there should be a moment (or age) at which we can safely stop follow-up after EET, because even if a recurrence occurs, the patient simply won’t live long enough to progress to symptomatic esophageal cancer. We think that this moment lies far below age 90. Keeping these patients under endoscopic follow-up has no direct clinical benefit, but instead, puts them at an unnecessary risk for complications, psychological stress, and needless hospital visits, and unnecessary costs.

For now, we think that even before talking money, the first step toward improving post-EET follow-up is developing more evidence-based follow-up protocols based on the recent long-term follow-up data that are available, to tailor surveillance on an individual basis, instead of the current one-size-fits-all approach. This protocol should be personalized based on an individual’s risk for recurrent dysplasia and life expectancy, and should also include a recommendation about when FU can safely be stopped.
